# Vaccination with Embryonic Stem Cells Protects against Lung Cancer: Is a Broad-Spectrum Prophylactic Vaccine against Cancer Possible?

**DOI:** 10.1371/journal.pone.0042289

**Published:** 2012-07-31

**Authors:** Kavitha Yaddanapudi, Robert A. Mitchell, Kalyani Putty, Sharon Willer, Rajesh K. Sharma, Jun Yan, Haribabu Bodduluri, John W. Eaton

**Affiliations:** 1 Molecular Targets Program, James Graham Brown Cancer Center, University of Louisville, Louisville, Kentucky, United States of America; 2 Tumor Immunobiology Program, James Graham Brown Cancer Center, University of Louisville, Louisville, Kentucky, United States of America; Istituto Superiore di Sanità, Italy

## Abstract

The antigenic similarity between tumors and embryos has been appreciated for many years and reflects the expression of embryonic gene products by cancer cells and/or cancer-initiating stem cells. Taking advantage of this similarity, we have tested a prophylactic lung cancer vaccine composed of allogeneic murine embryonic stem cells (ESC). Naïve C57BL/6 mice were vaccinated with ESC along with a source of granulocyte macrophage-colony stimulating factor (GM-CSF) in order to provide immunostimulatory adjuvant activity. Vaccinated mice were protected against subsequent challenge with implantable Lewis lung carcinoma (LLC). ESC-induced anti-tumor immunity was not due to a non-specific “allo-response” as vaccination with allogeneic murine embryonic fibroblasts did not protect against tumor outgrowth. Vaccine efficacy was associated with robust tumor-reactive primary and memory CD8^+^ T effector responses, Th1 cytokine response, higher intratumoral CD8^+^ T effector/CD4^+^CD25^+^Foxp3^+^ T regulatory cell ratio, and reduced myeloid derived suppressor cells in the spleen. Prevention of tumorigenesis was found to require a CD8-mediated cytotoxic T lymphocyte (CTL) response because *in vivo* depletion of CD8^+^ T lymphocytes completely abrogated the protective effect of vaccination. Importantly, this vaccination strategy also suppressed the development of lung cancer induced by the combination of carcinogen administration and chronic pulmonary inflammation. Further refinement of this novel vaccine strategy and identification of shared ESC/tumor antigens may lead to immunotherapeutic options for lung cancer patients and, perhaps more importantly, could represent a first step toward the development of prophylactic cancer vaccines.

## Introduction

One old theory concerning oncogenesis was that cancer arose from nests of embryonal stem cells, present in normal tissues and stimulated to grow by some kind of irritation [1]. Although this concept was largely ignored for over a century, there is now evidence that mutated, tissue-specific stem cells act as self renewing “cancer initiating cells”, responsible for the initiation of many malignancies [2]. In indirect support of this idea, there is abundant evidence that most solid tumor types express embryonic antigens to varying degrees [3], [4], [5]. For example, the ‘carcinoembryonic antigens’, first described in the mid-1960s, represent antigens shared by embryos and tumors of the digestive tract [6], [7]. Despite some success using these shared embryonic/tumor antigens as targets for immunotherapy in cancer patients, a persistent obstacle to these approaches has been that of tumor-induced immune tolerance [8].

An alternative immune-based anti-cancer strategy might be prophylactic vaccination against tumor antigens *prior* to the appearance of cancer – and the associated immune tolerance - but this approach is also fraught with problems. Although we now have some effective vaccines for cancers which arise from infectious agents (e.g., human papilloma virus [9]), cancers arise from ‘self’ and the majority of antigens displayed by tumor cells are also present on normal adult cells. However, it now appears that most tumors express certain embryonal antigens. Importantly, the expression of some embryonic antigens may end in the first or second trimester of pregnancy, well before the mammalian immune system determines ‘self’ versus ‘non-self’. Consequently, gene products expressed by both embryos and tumor cells may not be included in the ‘self’ repertoire and are therefore potentially immunogenic.

In the beginning of the 20th century, it was reported that prior injection of mice with fetal tissues led to rejection of transplantable tumors (reviewed in [10]). Klavins *et al.* later reported that antisera raised in rabbits against an emulsified whole human embryo (6–7 week) - adsorbed against adult human tissues - recognized a variety of human tumor types including skin, bronchial, renal, colonic, hepatic, lung and breast [11]. These observations support the concept that animals or humans immunized against embryonic material might be capable of recognizing and destroying neoplastic cells. Very recent studies describe the potential of ESC to prime anti-tumor immunity. These reports showed that pluripotent ESC induce modest delays in tumor growth in mouse models of transplantable colon carcinoma and lung cancer [12], [13].

Immunostimulatory cytokines, including GM-CSF, interleukin (IL)-2, IL-12, and interferon (IFN)-α have demonstrated significant anti-tumor effects. Among these, GM-CSF is one of the most potent and specific inducers of anti-tumor systemic immunity [14], [15]. GM-CSF mediates its effect by stimulating the activation of professional antigen-presenting cells (APCs), namely dendritic cells (DC) and macrophages. The APCs in turn, process and present tumor antigens to alert helper T cells and cytotoxic T lymphocytes (CTL) [16], [17]. Increased local production of GM-CSF by genetically modified tumor cells can induce specific anti-tumor cellular immunity both *in vitro* and *in vivo* [15], [17], [18], [19]. In the present study, we sought to improve upon the cancer immunotherapeutic potential of embryonic stem cell antigenic cross reactivity with malignant cells in an attempt to identify a more efficacious ESC-based anti-cancer vaccine.

Here, we report that vaccination with ESC in combination with a source of GM-CSF is effective in preventing implantable and carcinogen-induced lung tumors without detectable toxicity or signs of autoimmunity. The therapeutic efficacy of the ESC/GM-CSF combination vaccine was associated with robust tumor-specific primary and long-term memory CD8^+^ T effector responses, infiltration of CD8^+^ T cells into the tumor leading to increased intratumoral CD8^+^ T effector/T regulatory cell ratio and reduced myeloid derived suppressor cells (MDSCs) in the spleen. Collectively, our findings provide a strong rationale for further developing this novel form of vaccine as an immunotherapeutic strategy for the prevention of cancer.

## Results

### ESC vaccination prevents the outgrowth of an implanted lung adenocarcinoma

**Figure 1:**
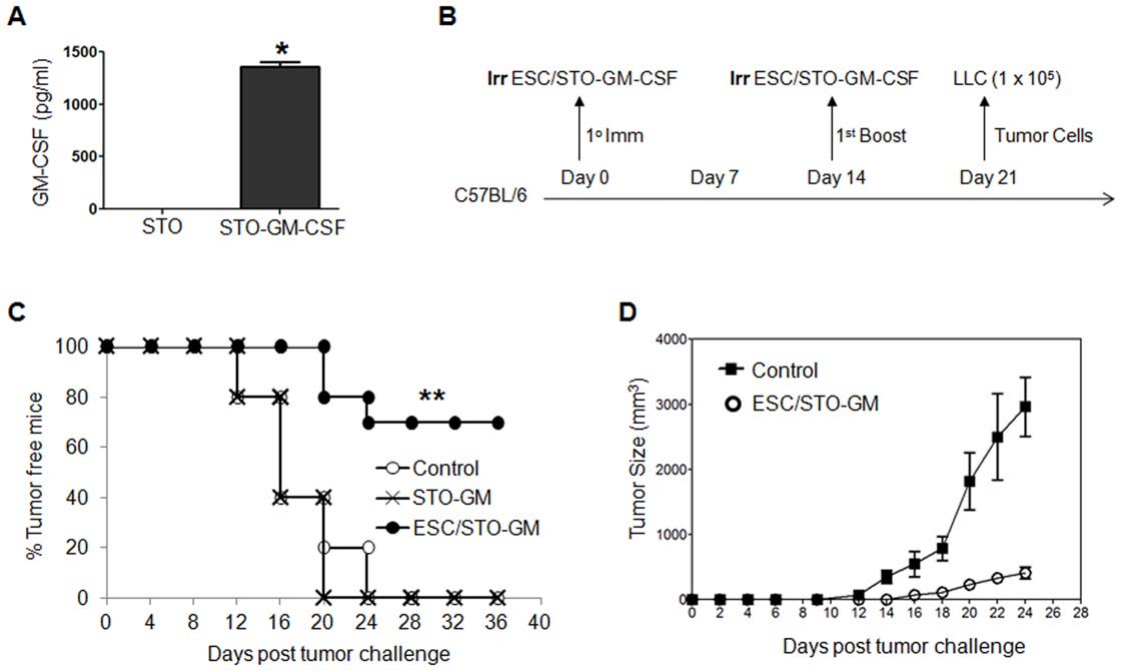
ESC vaccination prevents the outgrowth of an implanted lung adenocarcinoma. (**A**) Bar graph showing GM-CSF expression in non-transduced and retrovirally transduced STO fibroblasts. Error bars represent mean ± SD. *, p<0.05; relative to non-transduced STO cells; *t* test. (**B**) Scheme of immunization. Male C57BL/6 mice were immunized twice (days 0 and 14) with HBSS (control), or irradiated 1×10^6^ ESC+irradiated 1×10^6^ GM-CSF-expressing STO murine embryonic fibroblasts (STO-GM) s.c. in the right flank. Seven days after boost, mice were challenged with 1×10^5^ Lewis lung carcinoma cells (LLC) s.c. in the left flank. (**C**) C57BL/6 mice (10/group) were immunized twice (days 0 and 14) with HBSS (control), or irradiated 1×10^6^ ESC+irradiated 1×10^6^ STO-GM, or irradiated 1×10^6^ STO-GM cells alone s.c. in the right flank prior to s.c. challenge with LLC on day 21. Tumor growth was monitored daily in all animals until sacrifice due to tumors exceeding 5% of body weight. The vaccinated tumor free mice remained so for up to 4 months later with no overt signs of distress or autoimmunity. Results are representative of three independent experiments. **, *p*<0.001; relative to control group; log-rank test. (**D**). Tumor growth was measured by calipers every 2nd or 3rd day and tumor volumes were plotted as indicated. The data represent the average tumor volumes of 10 mice/control group and 3 mice/ESC/STO-GM group and are representative of three independent experiments. Error bars represent mean ± SEM.

Our initial studies attempted to assess the relative protective effect of ESC vaccine in the absence of any immunostimulatory adjuvants. Immunization of mice with irradiated ESC alone had no effect on the time of onset of Lewis lung carcinoma (LLC) tumor outgrowth but did result in a moderate decrease in initial tumor burden compared to tumors in non-immunized control mice (*data not shown*) consistent with prior studies [12], [13]. In order to enhance the effect of ESC vaccine by GM-CSF co-administration, we first attempted to over-express murine GM-CSF in the ES-D3 ES cell line using retroviral transduction as previously reported for B16 melanoma/GM-CSF vaccines [15]. However, due to difficulties in achieving appreciable GM-CSF expression using this approach - likely due to retroviral promoter element silencing in ESC [20] – we instead stably over-expressed GM-CSF in STO fibroblasts which are commonly used as feeder layers for ES-D3 cells. As shown in **Fig. 1A**, mock infected STO fibroblasts expressed negligible murine GM-CSF while GM-CSF retrovirally transduced STO fibroblasts express and secrete supra-physiologic amounts of the cytokine. Using a standard vaccination timing regimen (**Fig. 1B**), naïve C57BL/6 mice were immunized twice (days 0 and 14) with HBSS (control), irradiated ESC co-administered with STO fibroblasts expressing GM-CSF (ESC/STO-GM), or STO-GM cells alone. Seven days following the secondary immunization, mice were challenged with live LLC cells and monitored for tumor outgrowth as a function of time. As shown in **Fig. 1C**, vaccination of mice with ESC/STO-GM was 70% effective in preventing tumor outgrowth whereas all non-vaccinated control animals had developed palpable tumors by day 24 post-challenge. Importantly, irradiated STO-GM cells provided no protection against the outgrowth of LLC tumors (**Fig. 1C**) suggesting that the observed protection with ESC/STO-GM is not due to non-specific immune responses against allogeneic whole cell antigens. Finally, it is important to note that those LLC tumors that did develop in ESC/STO-GM vaccinated mice (n = 3) were significantly smaller and grew out at a much slower rate than those developing in non-vaccinated control mice (n = 10) (**Fig. 1D**). We next determined if our vaccination strategy provides protection against the outgrowth of other C57BL/6-derived tumors. Toward this end, we challenged ESC/STO-GM vaccinated mice with syngeneic B16F0 melanoma cell-line which originated from C57BL/6 mice and monitored tumor growth over time. As shown in **Figure S2**, ESC/STO-GM vaccination effectively reduced the size of melanoma tumors and also caused a significant delay in tumor outgrowh in comparison to control, non-vaccinated mice.

### ESC vaccination elicits tumor cell-specific CD8-dependent cytotoxic T lymphocyte response

**Figure 2:**
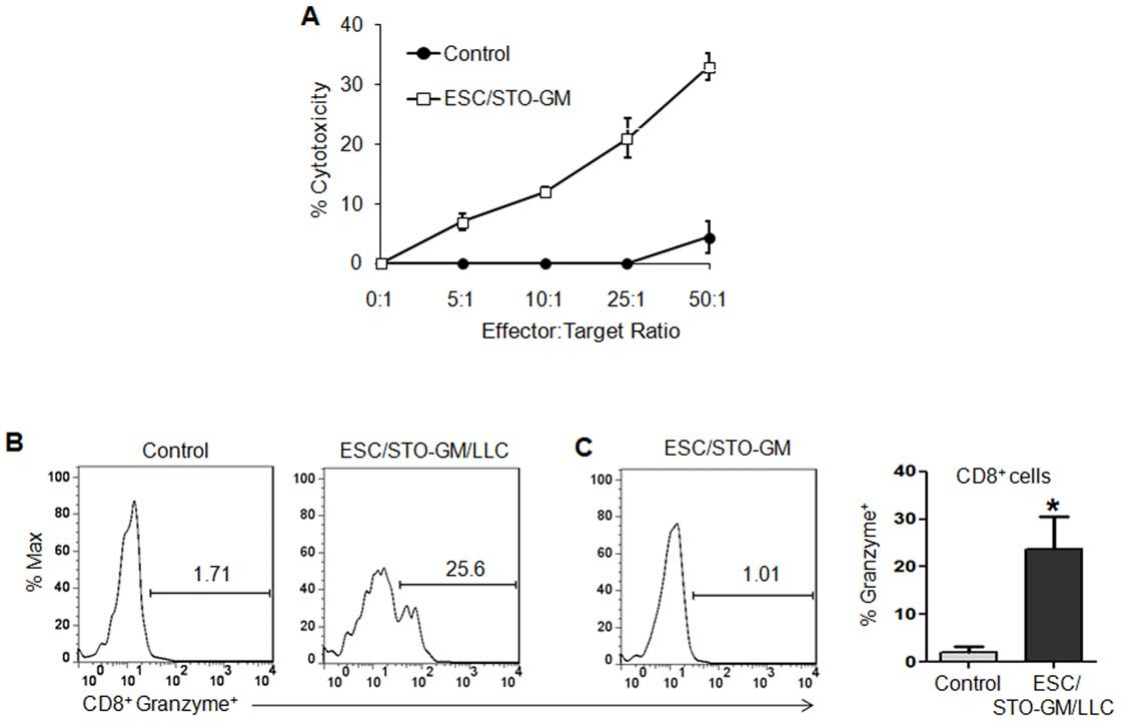
ESC vaccination elicits tumor cell-specific CD8-dependent cytotoxic response. (**A**) C57BL/6 mice (5/group) were immunized twice (days 0 and 14) with HBSS (control) or irradiated 1×10^6^ ESC+irradiated 1×10^6^ STO-GM cells s.c. in the right flank. Ten days after boost, mice were euthanized and spleens were removed. Splenocytes from vaccinated and control mice were added to wells pre-seeded with LLC cells and co-cultured for an additional 16 hours with the indicated effector-to-target cell ratios, with loss of the latter monitored in an Acea electrical impedance reader. Results shown represent four independent wells for each effector-to-target ratio and error bars represent mean ± SD. (**B–D**) C57BL/6 mice were immunized twice (days 0 and 14) with HBSS (control) or irradiated 1×10^6^ ESC+irradiated 1×10^6^ STO-GM cells s.c. in the right flank. Seven days after the last immunization, mice were challenged with 1×10^5^ LLC cells s.c. in the left flank. 18–21 days after tumor challenge, mice were euthanized and spleens were removed. Additionally, one set of mice were vaccinated with ESC/STO-GM alone and not challenged with tumor. Splenocytes from vaccinated/tumor challenged, vaccinated/non-tumor challenged and control mice were washed, Fc receptors were blocked, and stained for surface expression of CD8 and intracellular expression of Granzyme B and analyzed by flow cytometry. (**B, D**) Percentage of CD8^+^ cells expressing granzyme B in splenocytes obtained from vaccinated/tumor challenged and control mice (6/group). (**C**) Percentage of CD8^+^ cells expressing granzyme B in splenocytes obtained from vaccinated/non-tumor challenged mice (6/group). Results are expressed as percentages of gated CD8^+^ splenocytes (*, p<0.05; relative to control group; *t* test). Error bars represent mean ± SD.

Most successful cancer immunotherapies require a robust CD8-dependent CTL response [21], [22]. To investigate whether ESC/STO-GM vaccination elicits CD8 responses, splenocytes from ESC/STO-GM vaccinated and non-vaccinated (control) mice were assessed for *in vitro* tumor cell killing ten days after immunization. The results indicate the presence of a CTL response subsequent to ESC/STO-GM vaccination (**Fig. 2A**). Importantly, this killing is specific for tumor cells (and ESC) in that no cytotoxicity was observed against primary adult murine lung fibroblasts or STO fibroblasts (*data not shown*). We examined additional phenotypic markers on CD8^+^ splenocytes from ESC/STO-GM vaccinated and control mice. Granzyme B is a cytolytic molecule typically expressed by effector, but not naïve or memory, CD8^+^ splenocytes [23], [24]. Splenocytes were obtained from non-vaccinated/tumor challenged (control group), vaccinated/tumor challenged and vaccinated/non-tumor challenged mice and stained directly *ex vivo*, without any restimulation. As shown in **Fig. 2B**, only a very small fraction of CD8^+^ splenocytes isolated from unimmunized control mice expressed granzyme B, while 25.6% of CD8^+^ splenocytes from vaccinated/tumor challenged mice expressed this CTL effector (n = 6/group; *t* test, *p*<0.05; relative to control group; **Fig. 2B, D**). Furthermore, no differences in percentages of CD8^+^granzymeB^+^ cells were observed in splenocytes isolated from vaccinated mice that were not challenged with LLC cells in comparison to vaccinated/tumor challenged mice (**Fig. 2C**), providing additional evidence that the increased granzyme B response we observe in vaccinated/tumor challenged mice is specific to the cancer cells. As a further test of the importance of CD8-dependent CTL-mediated anti-tumor effects, CD4^+^ or CD8^+^ T lymphocytes were depleted *in vivo* using anti-CD4 or anti-CD8 monoclonal antibodies [25]. Whereas mice depleted of CD4^+^ lymphocytes were at least partially protected against the outgrowth of LLC, CD8^+^ T cell depletion completely abrogated the protective effect of ESC/STO-GM vaccination (**Table 1**).

**Table 1:** CD8^+^ T cell depletion abrogates vaccine-mediated protection from the outgrowth of implanted Lewis lung carcinoma.

Treatment	Mice w/tumor/Total # of mice
No vaccine, No treatment	5/5
ESC/STO-GM+Isotype mAb	0/5
ESC/STO-GM+CD4 mAb	2/5
ESC/STO-GM+CD8 mAb	5/5

Following vaccination (0 and 14 days), the mice (5/group) were injected with anti-CD4, anti-CD8 or isotype control IgG monoclonal antibodies immediately preceding tumor challenge (day 21) and every 4 days subsequently for a total of five injections.

### ESC vaccination induces tumor cell-specific, Th1-mediated cytokine response in CD8^+^ T cells

**Figure 3:**
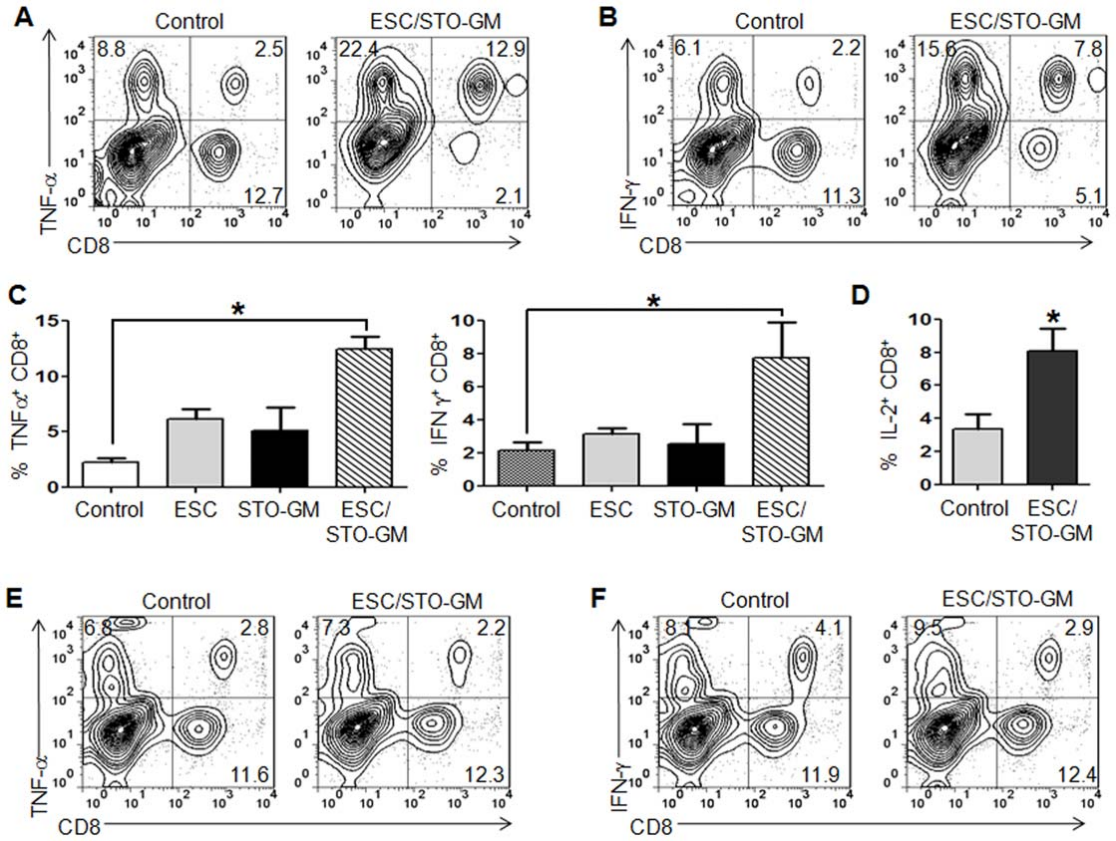
ESC vaccination induces tumor cell-specific, Th1-mediated cytokine response in CD8^+^ T cells. C57BL/6 mice (6/group) were immunized twice (days 0 and 14) with HBSS (control) or irradiated 1×10^6^ ESC alone, or irradiated 1×10^6^ ESC+irradiated 1×10^6^ STO-GM, or irradiated 1×10^6^ STO-GM cells alone, s.c. in the right flank. Ten days after the boost, mice were euthanized and spleens were removed. Splenocytes from vaccinated and control mice were co-cultured with LLC lysate (50 µg/ml) for an additional 4 days. Effectors were harvested and stimulated for 4 hours with PMA (50 ng/ml) and ionomycin (500 ng/ml) in the presence of Brefeldin A (1 µl/ml). After restimulation, effectors were harvested, Fc receptors were blocked, and stained for surface expression of CD4, CD8 and intracellular expression of cytokines and analyzed by flow cytometry. (**A, B**) Dot plots showing TNF-α and IFN-γ expression in CD8^+^ cells in splenocyte cultures obtained from control and ESC/STO-GM vaccinated mice. Numbers in quadrants represent the percentages of each subpopulation. (**C, D**) Bar graphs showing percentages of CD8^+^TNF-α^+^, CD8^+^IFN-γ^+^, and CD8^+^IL2^+^ cells in splenocyte cultures derived from control, ESC alone, STO-GM alone and ESC/STO-GM vaccinated mice. Results are expressed as percentages of total cells. *, p<0.05; relative to control group; ANOVA. (**E, F**) Unstimulated spleen cells from vaccinated and control mice were directly treated with PMA/ionomycin/Brefeldin A and stained for intracellular cytokine expression. Two independent cell culture assays were performed with cells isolated from 6 mice per group; data from one representative assay is shown. Error bars represent mean ± SD.

We next determined the ability of CD8^+^ T cells from vaccinated mice to produce effector cytokines required for effective anti-tumoral cytolytic activity. In response to re-stimulation with LLC cell lysate, a significantly higher frequency of IFN-γ, TNF-α and IL-2 producing CD8^+^ splenocytes were obtained from ESC/STO-GM vaccinated mice when compared with the non-vaccinated control group (n = 6/group; ANOVA, *p*<0.05; relative to control group; **Fig. 3A–D**). In the absence of LLC re-stimulation, no increase in cytokine production was observed in CD8^+^ splenocytes from vaccinated mice when compared to unstimulated, control non-vaccinated mice (**Fig. 3E, F**). When analyzing the independent contributions of ESC and STO-GM immunizations to CD8^+^ T cell effector functions, we observed that ESC alone or STO-GM treatments alone failed to induce a significant increase in the percentages of splenic CD8^+^TNF-α^+^ and CD8^+^IFNγ^+^ cells in comparison to control non-vaccinated mice (**Fig. 3C**). To determine if ESC/STO-GM vaccinated mice induced similar CD8^+^ T effector responses to other C57BL/6 derived tumor cells, we performed *in vitro* cytokine analysis with lysates derived from B16F0 melanoma cells. As expected, in response to re-stimulation with B16 cell lysate, we observed a significant increase in the frequency of IFN-γ and TNF-α producing CD8^+^ splenocytes from ESC/STO-GM vaccinated mice in comparison to restimulated splenocyte cultures obtained from non-vaccinated control group (**Figure S2**).

### ESC vaccination reduces myeloid-derived suppressor cells but does not alter T regulatory cells in the spleen

**Figure 4:**
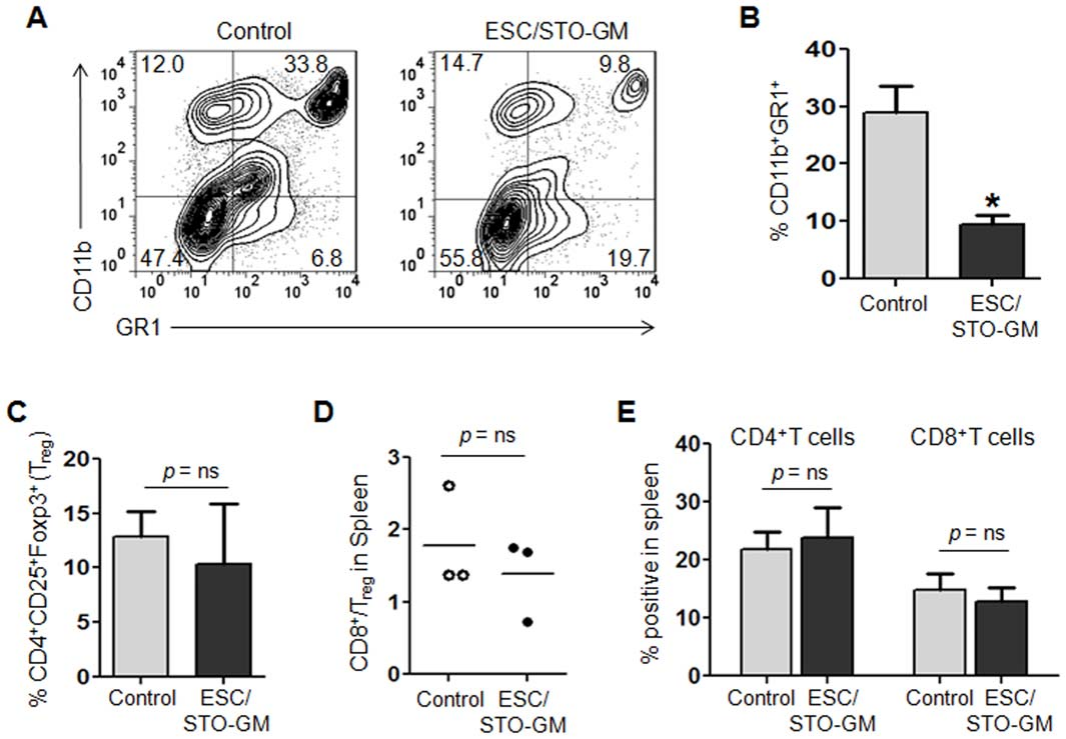
ESC vaccination reduces myeloid-derived suppressor cells but does not alter T regulatory cells in the spleen. C57BL/6 mice were immunized twice (days 0 and 14) with HBSS (control) or irradiated 1×10^6^ ESC+irradiated 1×10^6^ STO-GM cells s.c. in the right flank. Seven days after the last immunization, mice were challenged with 1×10^5^ LLC cells s.c. in the left flank. 18–21 days after tumor challenge, mice were euthanized and spleens were removed. Splenocytes from vaccinated and control mice were washed, Fc receptors were blocked, and stained for surface expression of different markers and analyzed by flow cytometry. (**A**) Dot plots showing percentages of splenic CD11b^+^GR1^+^ myeloid-derived suppressor cells (MDSCs) in control and ESC/STO-GM vaccinated mice. Numbers in quadrants represent the percentages of each subpopulation. (**B**) Bar graphs showing percentages of CD11b^+^GR1^+^ MDSCs in splenocytes obtained from control and ESC/STO-GM vaccinated mice. Results are expressed as percentages of total cells. *, p<0.05; relative to control group; *t* test. (**C**) Bar graphs showing percentages of CD4^+^CD25^+^Foxp3^+^ T regulatory cells (T_reg_) in splenocytes obtained from control and ESC/STO-GM vaccinated mice. Results are expressed as percentages of total cells. (**D**) The ratio of CD8^+^Foxp3^−^ to CD4^+^CD25^+^Foxp3^+^ T_reg_ cells was calculated and compared in splenocytes obtained from control and ESC/STO-GM vaccinated mice. (**E**) Bar graph showing percentages of CD4^+^ T and CD8^+^ T cells in splenocytes obtained from control and ESC/STO-GM vaccinated mice. Results are expressed as percentages of total cells. Three independent analyses were performed with cells isolated from 5 mice per group; data from one representative assay is shown. Error bars represent mean ± SD.

To further study the immunomodulatory effects of ESC/STO-GM vaccination, we analyzed the phenotype of splenocytes by flow cytometry. It has been demonstrated that CD11b^+^Gr1^+^ myeloid–derived suppressor cells (MDSCs) and CD4^+^CD25^+^Foxp3^+^ T regulatory cells (T_regs_) are the two suppressor populations that impede the anti-tumoral effector responses in the spleen [26], [27]. We found that the percentage of MDSCs was significantly decreased from 33.8% to 9.8% in the spleens of mice vaccinated with ESC/STO-GM and challenged with LLC cells when compared with non-vaccinated, LLC challenged control mice (n = 5/group; *t* test, *p*<0.05; relative to control group; **Fig. 4A, B**). ESC/STO-GM vaccination, however, did not reduce the percentages of splenic T_regs_ (**Fig. 4C**) or induce any change in the ratio of CD8^+^ T cells to T_regs_ in the spleen (**Fig. 4D**). Furthermore, we did not observe any significant differences in the percentages of CD4^+^ and CD8^+^ T cells (**Fig. 4E**) or in their absolute number (*not shown*) in the spleens from vaccinated and control mice.

### ESC vaccination increases the ratio of CD8^+^ T effector cells to T_regs_ in the tumor

**Figure 5:**
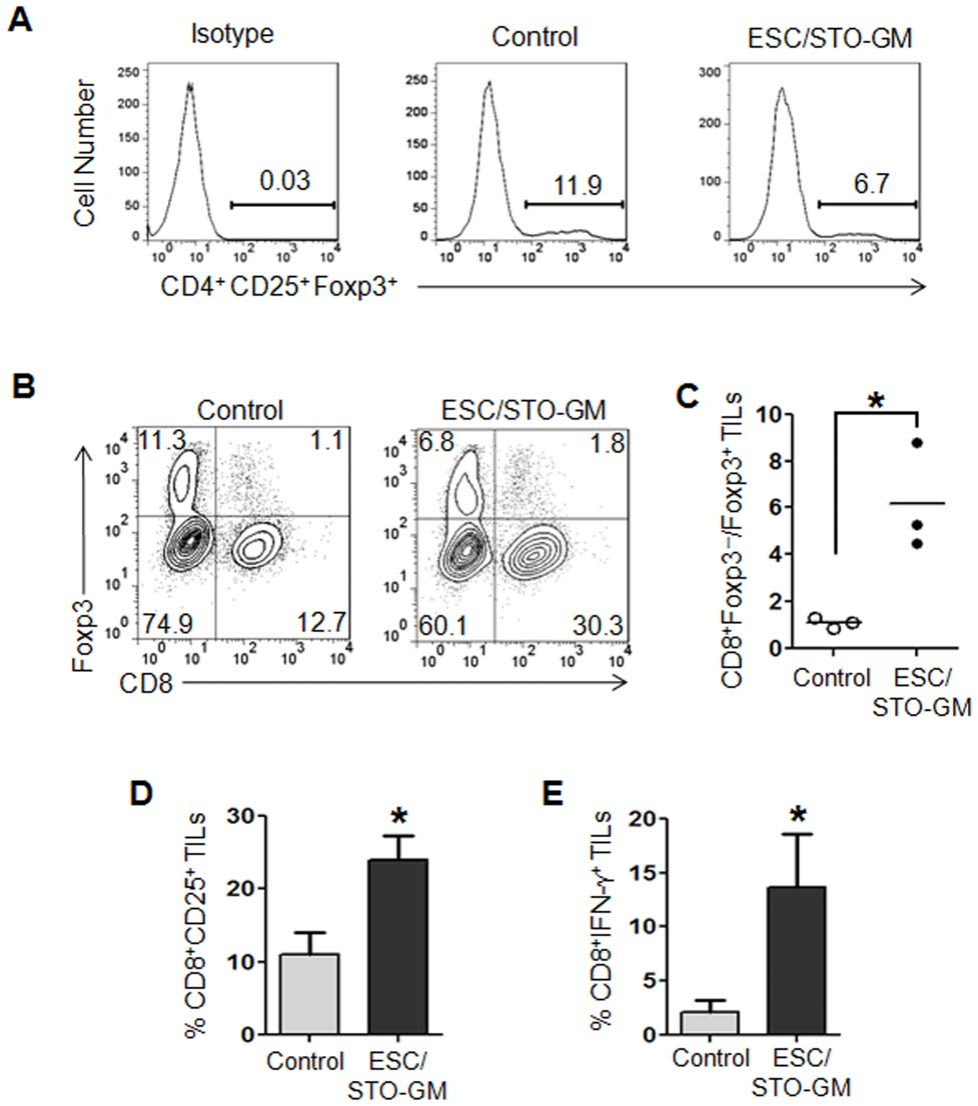
ESC vaccination increases the ratio of effector CD8^+^ T cells to T_regs_ in the tumor. C57BL/6 mice were immunized twice (days 0 and 14) with HBSS (control) or irradiated 1×10^6^ ESC+irradiated 1×10^6^ STO-GM cells s.c. in the right flank. Seven days after boost, mice were challenged with 1×10^5^ Lewis lung carcinoma cells s.c. in the left flank. 18–21 days after tumor challenge, tumor-infiltrating cells were harvested from control and ESC/STO-GM vaccinated mice and analyzed by flow cytometry. (**A**) Histograms showing the percentages of CD4^+^CD25^+^Foxp3^+^ T_regs_ in CD45.2^+^ tumor infiltrating cells obtained from control and ESC/STO-GM vaccinated mice. (**B**) Dot plots showing the percentages of CD8^+^ and Foxp3^+^ sub-populations in CD45.2^+^ tumor infiltrating cells. (**C**) Bar graph showing the ratio of CD8^+^ Foxp3^−^ to CD8^−^Foxp3^+^ cells in 1 of 2 representative experiments with 3 independently analyzed mice/group. *, p<0.05; relative to control group; *t* test. (**D, E**) ESC vaccination increases the frequency of functional CD8^+^ T cells in tumors. (**D**) Bar graph showing the percentages of CD25^+^CD8^+^ in CD45.2^+^ tumor infiltrating cells obtained from control and ESC/STO-GM vaccinated mice. (**E**) Tumor infiltrating cells were restimulated with PMA and ionomycin and analyzed for the expression of intracellular IFN-γ. Bar graphs showing percentages of CD8^+^IFN-γ^+^ in tumor infiltrating cells from control and ESC/STO-GM vaccinated mice. Results are expressed as percentages of total cells. The data represent results from 2 independent experiments with 3 mice/group. *, p<0.05; relative to control group; *t* test. Error bars represent mean ± SD.

Our results indicate that the ESC/STO-GM-induced anti-tumor effector function is predominantly mediated by CD8^+^ T cells. We therefore studied the impact of this vaccine regimen on the function of intra-tumoral CD8^+^ cells and their interaction with T_regs_. Utilizing the small numbers of ESC/STO-GM vaccinated mice that did develop LLC lesions – albeit in a delayed fashion (**Fig. 1C, D**), tumors from controls and vaccinated mice were harvested and used to investigate the subset profiles of tumor-infiltrating lymphocytes (TILs). Flow cytometric analyses of CD45.2^+^ TILs revealed a decrease in the percentage of CD4^+^CD25^+^Foxp3^+^ T_regs_ in tumor infiltrates from vaccinated mice *versus* controls (**Fig. 5A**). Although we still observed the presence of T_regs_ in these tumors, the ratio of CD8^+^ T cells to T_regs_ was significantly increased in the tumor infiltrates from ESC/STO-GM vaccinated mice (n = 3/group; *t* test, *p*<0.05; relative to control group; **Fig. 5B, C**). To assess the quality of CD8^+^ T cell tumor infiltrates following ESC/STO-GM vaccination, we analyzed the expression of the activation marker CD25 and the effector cytokine IFN-γ. CD8^+^ cells in the ESC/STO-GM tumor infiltrates had elevated CD25 expression and were capable of producing higher amounts of IFN-γ in response to re-stimulation with PMA/ionomycin (n = 3/group; *t* test, *p*<0.05; relative to control group; **Fig. 5D, E**). Thus, the ESC and STO-GM combination vaccine significantly increases the ratio of CD8^+^ T cells to T_regs_ and the percentages of CD8^+^CD25^+^ and CD8^+^IFN-γ^+^ effector cells within the tumors and are suggestive of efficient vaccine-induced, tumor-reactive immune system priming.

### ESC vaccination-induced CD8^+^ T cell-mediated effector responses are maintained in long-term surviving animals

**Figure 6:**
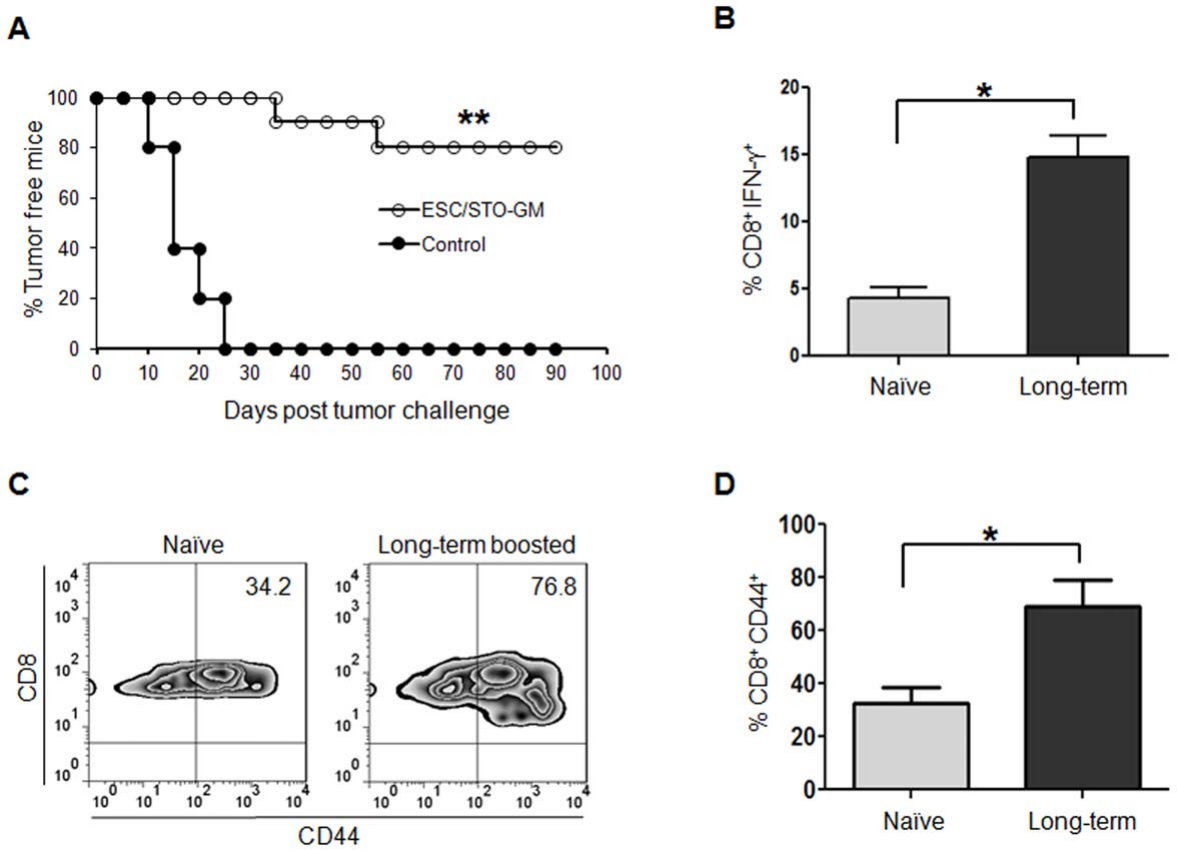
ESC vaccination-induced CD8^+^ T-effector responses are maintained in long-term surviving animals. Long-term surviving mice from the ESC/STO-GM group were re-challenged s.c with 1×10^5^ LLC cells 60 days after initial tumor inoculation. (**A**) Tumor growth was monitored daily in all animals until sacrifice due to tumors exceeding 5% of body weight. Results represent a summation of two independent experiments with 10 mice/group. **, p<0.001; relative to naïve control; log-rank test. (**B–D**) Spleens were isolated from vaccinated survivors and control naïve mice 10 days after the tumor injection. (**B**) Splenocytes were harvested and stimulated for 4 hours with PMA (50 ng/ml) and ionomycin (500 ng/ml) in the presence of Brefeldin A (1 µl/ml). After restimulation, cells were harvested, Fc receptors were blocked, and stained for intracellular expression of cytokines, and analyzed by flow cytometry. Bar graphs showing percentage of intracellular IFN-γ expression in CD8^+^ cells (*, *p*<0.05; relative to naïve control; *t* test). Results are expressed as percentages of total cells. (**C**) Dot plots showing the percentages of CD44^+^ and CD8^+^ cells in splenocytes derived from long-term surviving ESC vaccinated and control naïve mice. Numbers in the quadrants represent percentages of each subpopulation. (**D**) Bar graph showing the percentages of CD44^+^CD8^+^ cells (*, *p*<0.05; relative to naïve control; *t* test). Results are expressed as percentages of total cells. Data for B–D are representative of two independent experiments with 4 mice/group. Error bars represent mean ± SD.

We next tested the efficacy of our ESC vaccination strategy in the maintenance of CD8^+^ T-cell memory pool and generation of secondary responses using mice that had been successfully vaccinated/protected 60 days earlier. Controls included naïve, non-vaccinated mice that were challenged with LLC cells. 80% of mice previously vaccinated with ESC/STO-GM had not developed tumors 90 days after tumor re-inoculation, while 0% of age-matched controls remained tumor free (n = 10/group; log-rank test, *p*<0.001; relative to naïve control group; **Fig. 6A**). Importantly, vaccinated animals with eradicated LLC tumors retained long-term immunologic memory as assessed by increased production of IFN-γ by splenic CD8^+^ cells (n = 4 mice/group; *t* test, *p*<0.05; relative to naïve control group; **Fig. 6B**). Furthermore, we observed a significant increase in splenic CD8^+^CD44^hi^ memory cells in long-term vaccinated mice re-challenged with LLC tumor cells when compared with naïve tumor challenged mice. (n = 4 mice/group; *t* test, *p*<0.05; relative to naïve control group; **Fig. 6C, D**).

### ESC vaccination suppresses 3-methylcholanthrene initiated, butylated hydroxytoluene promoted lung carcinogenesis

**Figure 7:**
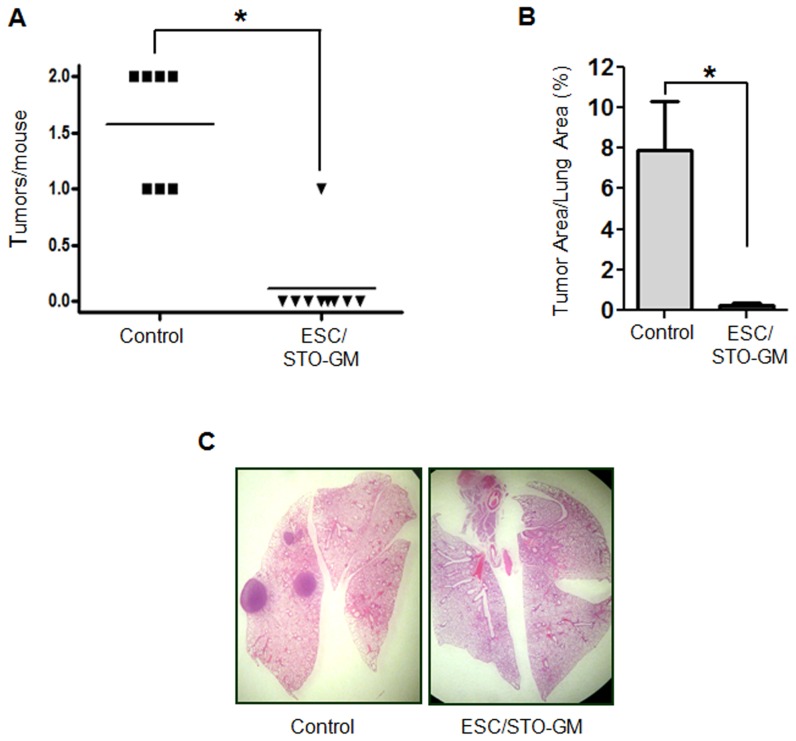
ESC vaccination suppresses 3-methylcholanthrene initiated, butylated hydroxytoluene promoted lung carcinogenesis. (**A**) Eighteen weeks after administration of the single dose of 3-methylcholanthrene followed by repetitive doses of BHT, lungs from euthanized Balb/c control and ESC/STO-GM vaccinated mice (8/group) were resected and inflated at a pressure of 15 cm with 4% buffered formalin. Surface tumors were enumerated by inspection under 5× magnification. *, p<0.05; relative to control group; *t* test. (**B**) The percentage of total lung area taken up by adenocarcinomas was quantified from measurements on H&E stained serial sections of lungs from three animals in each group (sections examined were 100 µm apart). *, p<0.05; relative to control group; *t* test. Error bars represent mean ± SD. (**C**) Representative H&E stained sections from control or ESC/STO-GM vaccinated mice (8/group) were photographed under low power (5×).

Prevention of the outgrowth of implantable lung tumors using this vaccination strategy does not predict efficacy in *in vivo* pulmonary tumorigenesis. To test the latter, we utilized a mouse model involving carcinogen administration followed by chronic pulmonary inflammation which is thought to mimic smoking-associated lung carcinogenesis [28]. Male Balb/c mice were exposed to a bolus of 3-methylcholanthrene (experimental week 1) and repetitive administration of butylated hydroxytoluene (BHT; weeks 3–8). These animals were then vaccinated (weeks 4, 6 and 8) with either HBSS alone or ESC/STO-GM. In this model, multiple lung tumors predictably form within four to six months following initiation. 18 weeks after the initial dose of 3-methylcholanthrene, lungs were harvested and fixed. Initial gross examination of the lungs revealed that control vaccinated mouse lungs all had surface tumors with an average of 1.6 large lesions per mouse (**Fig. 7A**). In contrast, only 1/8 mice vaccinated with ESC/STO-GM had a visible surface tumor giving an average of ∼0.2 lung tumors/mouse (n = 8; *t* test, *p*<0.05; relative to control group; **Fig. 7A**). Total lung tumor mass was further evaluated in 3 animals of each group by examination of serial sections of the lungs. It is important to note that vaccinated animals chosen for subsequent lung serial section analyses included the one mouse with a visible surface tumor. As shown in **Fig. 7B**, ESC/STO-GM vaccinated mice had a dramatically smaller percentage of tumor-bearing lung area compared to unvaccinated controls (*t* test, *p*<0.05; relative to control group). Typical whole lung sections from these animals showed a striking difference between mock-vaccinated carcinogen-treated mice versus those receiving ESC/STO-GM vaccines (**Fig. 7C**). Numerous large adenocarcinomas were detected in non-vaccinated control mouse lung sections (**Fig. 7C**, left panel), whereas vaccinated animals had a nearly complete absence of any lesions (**Fig. 7C**, right panel).

## Discussion

The development of non-toxic, potent immunomodulatory agents is crucial to the success of prophylactic vaccines. Overall, our results support the idea that antigenic similarities between embryonic and malignant cells may be useful in the design of prophylactic vaccines to prevent lung tumorigenesis. In fact, in one regard this may not be too surprising. Several recent studies indicate that tumors may arise as a result of aberrant proliferation of organ-specific, self-renewing stem cells that are both necessary and sufficient for tumor initiation [2]. Examples of malignancies reported to have these ‘cancer initiating cells’ include solid tumors such as breast, brain, lung, prostate and pancreas [29], [30], [31], [32], [33]. It is possible - but far from certain - that ESC vaccination may selectively target this small subset of highly tumorigenic stem-like cells.

Irradiated, allogeneic murine ESC were used in our vaccination strategy because allogeneic whole cell vaccines are reportedly more effective as immunogens than autologous cell vaccines [34]. Furthermore, syngeneic live ESC might form teratomas or embryomas. To promote a more robust response to immunization, we co-administered ESC with STO mouse embryonic fibroblasts retrovirally infected with a GM-CSF expression construct. These STO cells are frequently used as a feeder layer for ESC culture. STO cells expressing GM-CSF were used - rather than ESC expressing GM-CSF – because of retroviral silencing by ESC [20], coupled with the fact that long term exposure to GM-CSF has been reported to force the differentiation of ESC [35].

ESC vaccination was effective in reducing carcinogen-induced lung tumorigenesis and preventing LLC implantable tumor development in ∼70–80% of mice with tumor-free survival over a 90-day observation period. Vaccinated mice which remained tumor free on first challenge did not develop tumors when re-challenged 60 days later with live LLC cells, demonstrating the existence of long-term immunological memory. The long-term memory response was substantiated by an increased pool of memory CD8^+^ T cells and enhanced Th1 cytokine responses in long-term surviving mice with effective vaccination. The anti-tumor efficacy of the ESC/STO-GM vaccine also correlated with significantly higher intra-tumoral CD8^+^ T/CD4^+^CD25^+^Foxp3^+^ T_regs_ ratio. T_regs_ represent a major barrier to effective cancer immunotherapy, with high abundance of T_regs_ in tumors associated with poor prognosis of cancer patients [36], whereas a high T_eff_/T_reg_ cell ratio positively correlates with successful therapies [37], [38].

Although the nature of the anti-tumor effects of ESC vaccination is still not known, it is possible that it may involve expansion of an effective pool of cytotoxic T lymphocytes as well as increased trafficking and entry of CD8^+^ T cells into the tumor microenvironment. In contrast to the situation within the tumor, ESC and GM-CSF combination vaccine did not alter T_eff_/T_reg_ ratios in the periphery. However, we did observe a significant decrease in the percentage of MDSCs in the spleens of vaccinated/tumor challenged mice when compared to non-vaccinated/tumor challenged control mice. The accumulation of MDSCs in lymphoid organs has been observed in tumor-bearing individuals and often correlates with an immunosuppressive phenotype and increased tumor burden [27], [39], [40], [41]. Our results are consistent with an earlier study showing that ESC based vaccines reduce splenic MDSCs in a mouse model of transplantable colon carcinoma [12].

The impact of the ESC/STO-GM vaccine on the CD8^+^ compartment may be either indirect or direct. Our *in vivo* depletion studies suggest that the preventive effect of ESC vaccination involves CD8^+^ T cells rather than CD4^+^ T cells. Although enhanced CD4^+^ T cell help appears not to be required for mounting primary immune responses, it may be critical for the generation and maintenance of long-term memory and recall responses [42], [43] in the vaccinated animals.

There is precedent for the idea that vaccination with embryonic/fetal material might confer cross-immunity to cancer. In fact, one hundred years ago, Frederick Schöne, an assistant of Paul Erlich, reported that vaccination of mice with preparations of early fetal material would partially prevent the outgrowth of transplanted tumors [44]. Many years after Schöne's seminal observation, a number of other investigators variously reported that immunization of animals with early embryonic material (usually irradiated cells or tissue from syngeneic donors) would, to some extent, suppress or prevent the growth of transplantable tumors [45]. However, the anti-tumor effects tended to be weak and poorly reproducible. Following an earlier informal report of our results [46], Li *et al* showed that human ESC were able to induce a delay in tumor growth in a mouse model of transplantable colon carcinoma without evidence of any autoimmune disease [12]. The vaccination strategy we have employed here provides far more potent and consistent protection against tumor outgrowth than observed in these earlier reports [12], [13]. We speculate that this is due to our use of a combination vaccine approach comprising allogeneic ESC and cells producing the immunostimulatory cytokine, GM-CSF.

It is likely that the protection against lung cancer afforded by ESC/STO-GM vaccination involves a number of shared antigens. If so, this may represent a strength of this vaccination approach in as much as immune recognition of multiple antigens would make it less likely for nascent tumor cells to escape immune detection and destruction. The use of whole cells as the antigenic component of a vaccine alleviates the concerns related to the limitations of targeting single antigens to generate anti-tumor responses that include: (i) saturation of immune response due to antigen exhaustion, (ii) lesser magnitude and duration of immune response towards a single epitope as compared to collective responses to multiple epitopes and (iii) higher possibility of immune-edited escape variants. LLC is a rapidly growing transplantable tumor model and, therefore, antigen escape variants due to immunological pressure may not occur within the time frame of the experiment. However, this may be totally different in a spontaneous tumor setting, where immunological pressure may likely to give rise to antigen-loss variants [47], [48], [49]. Therefore, a whole cell based ESC/STO-GM vaccine may have better efficacy even in a spontaneous setting due to the availability of multiple CD8^+^ T cell epitopes. Consistent with this notion, we demonstrated potent anti-tumor responses in a carcinogen-induced lung cancer model in mice upon vaccine administration. Our vaccination strategy appears to be safe and non-toxic, with vaccinated animals showing no signs of autoimmune disease and no evidence of outgrowth of teratomas or embryomas at the site of vaccination.

The development of non-toxic immunotherapeutic agents that not only activate the effector arm of the immune system but also overcome various immune evasion mechanisms employed by the progressing tumors may be the key to the success of anti-cancer vaccines. Our results raise the exciting possibility of developing a prophylactic vaccine capable of preventing the appearance of various types of cancers in humans, especially those with hereditary, chronological or environmental predispositions to neoplastic disease.

## Materials and Methods

### Mice

#### Ethics Statement

All mice were handled in accordance with the Association for Assessment and Accreditation of Laboratory Animals Care international guidelines, with the approval of the Institutional Animal Care and Use Committee at University of Louisville. The University of Louisville IACUC reviewed and approved this study under ID # 09021.

Wild-type male C57BL/6 and BALB/c mice were obtained from The Jackson Laboratory (Bar Harbor, ME, http://www.jax.org) or Harlan Laboratories (Dublin, VA). Mice were maintained at the University of Louisville Health Center using standard guidelines. Mice were used at 6–8 weeks of age.

### Cell lines

As a vaccine, we employed the murine embryonic stem cell (ESC) line, ES-D3 (ATCC CRL-11632), derived from 129/Sv mice (expressing MHC class II I-E). ESC were cultured under 5% CO2 in Dulbecco's modified eagle's medium (DMEM) supplemented with 15% ES Cell Qualified fetal bovine serum, 50 U/ml penicillin, 50 µg/ml streptomycin, 0.1 mM non-essential amino acids, 0.1 mM β-mercaptoethanol and 2 mM L-glutamine (all from GIBCO, Invitrogen Corporation, Grand Island, NY) under standard conditions. No feeder layer was used, but leukemia inhibitory factor (Chemicon, Temecula, CA) was added at a concentration of 80 units/ml (500 pM) to prevent differentiation of the ESC during culture. ESC were periodically evaluated using anti-SSEA-1 (MC-480, Developmental Studies Hybridoma Bank, Iowa City, IA) and with BD Stemflow™ human and mouse pluripotent stem cell analysis kit (BD Biosciences, San Jose, CA) to ensure retention of an undifferentiated state (**Figure S1**).

### Vaccinations

Stem cells were removed from the plate with enzyme-free cell dissociation solution (Specialty Media, Phillipsburg, NJ), washed twice in sterile Hank's buffered salt solution (HBSS) and suspended in HBSS at a concentration of 10×10^6^/ml. The cells were injected subcutaneously (s.c), 1×10^6^ per inoculation, in the right flank of male C57BL/6 mice (which lack MHC class II I-E expression). Murine fibroblasts expressing GM-CSF (1×10^6^ per inoculation) were co-administered with the ESC (ESC/STO-GM vaccine). STO fibroblast cell line (ATCC # CRL-1503) was infected in culture with a replication-defective retrovirus expressing murine GM-CSF (a gift from Dr. Glenn Dranoff, Dana Farber Cancer Institute) and maintained and processed under the same conditions as the ESC. GM-CSF production by these cells was ensured by ELISA measurements on cell supernatants (R & D Systems, Minneapolis, MN). ESC and STO-GM cells were irradiated (15 Gy) before immunization. In all cases, we administered a primary vaccination on day 0 and a boost on day 14 followed by tumor challenge on day 21.

### Implantation of Lewis lung carcinoma or B16F0 melanoma and evaluation of anti-tumor responses

Lewis lung carcinoma (LLC) and B16 melanoma cells (originally derived from C57BL/6 mice) were cultured under standard conditions in DMEM supplemented with 15% ES Cell Qualified fetal bovine serum as above. The cells were lifted with trypsin/EDTA, washed twice in sterile PBS and suspended at a concentration of 1×10^6^/ml in HBSS immediately prior to inoculation. Tumor cells (1×10^5^) were administered by subcutaneous inoculation in the mid-left femoral region of male 6–8 week old C57BL/6 mice. Tumor growth was monitored every 3 days using digital calipers to measure both the longitudinal and transverse diameters (in mm). Mice were also monitored for general health indicators such as overall behavior, feeding, body weight and appearance of fur, after immunization. Animals bearing tumors were euthanized when tumors reached a size of 15 mm in diameter or earlier if tumors ulcerated or animals showed signs of discomfort. For long-term memory responses; surviving mice were rechallenged with LLC cells (1×10^5^) on day 60 after the initial tumor cell injections.

### Cytotoxicity assay

*In vitro* cytotoxicity of splenocytes from ESC/STO-GM-vaccinated mice against LLC cells was analyzed using measurements of changes in the electrical impedence exerted by viable target cells adherent to 16-well plates (Acea Biosciences, Inc.; San Diego, CA). Briefly, 1×10^4^ LLC cells were placed into the wells of Acea 16-well plates, maintained in DMEM medium and acclimated to the environment within the plate for 24 hours. Primary splenocytes isolated from non-vaccinated and vaccinated mice were then added to the wells to achieve effector-to-target cell ratios of between 0∶1 and 50∶1. Cells were incubated in the real-time cell electronic sensing (RT-CES) instrument (Acea Biosciences) maintained at 37°C in a humidified 5% CO_2_ incubator for 16 hours. Cytotoxicity was determined by measuring the relative decrease in current impedance among wells with no effector cells compared to those with various effector∶target ratios.

### Antibodies

All antibodies except anti-granzyme B, were purchased from either BD Biosciences (San Jose, CA) or eBioscience (San Diego, CA). Anti-granzyme B was purchased from Caltag (Invitrogen, Carlsbad, CA).

### Flow cytometric analysis

Single cell suspensions from spleen were stained with relevant antibodies (CD3, CD4, CD8, CD44, CD62L, CD69, CD25, CD43, CD11b, Ly6G, Ly6C) for 30 min after blocking with CD16/CD32 antibody (2.4G2; BD Biosciences, San Jose, CA) for 15 min at 4°C. After washing, cell surface and intracellular stained cells were analyzed on a FACSCalibur (Becton Dickinson and Company, Franklin Lakes, NJ) and results were analyzed with FlowJo software (TreeStar, Inc., Ashland, OR).

### Intracellular cytokine staining

Spleens were isolated from different treatment groups 10 days after the last vaccination. Splenocytes were stimulated with LLC or B16 lysate (50 µg/ml) for 4 days. For TNF-α, IFN-γ and IL-2 production, cells (effectors) were harvested and incubated for 4 hours with PMA (50 ng/ml) and ionomycin (500 ng/ml) in the presence of Golgiplug (containing brefeldin A; BD PharMingen, San Jose, CA) at a dilution of 1 µl/ml. After restimulation, effectors were harvested, Fc receptors were blocked, and stained for surface expression of CD4, CD8 and intracellular expression of cytokines using Cytofix/Cytoperm kit according to the manufacturer's instructions (BD Pharmingen) and analyzed by flow cytometry. Additionally, unstimulated spleen cells from vaccinated and control mice were directly treated with PMA/ionomycin/Brefeldin A and stained for intracellular cytokine expression using the same procedure as described above. For granzyme B staining, splenocytes were stained directly *ex vivo*, without any restimulation. After washing, cells were subjected to surface staining with CD16/32 antibody followed by anti-CD8 and intracellular staining using anti-granzyme B-PE antibodies. Cells were fixed in 2% paraformaldehyde/phosphate-buffered saline and analyzed by flow cytometry.

### Analysis of tumor-infiltrating T cells

Vaccinated and control mice bearing LLC tumors were euthanized 18–21 days after tumor challenge. Solid tumors were dissected and chopped into small pieces before incubation with a mixture of enzymes dissolved in HBSS (400 U/ml collagenase type IV, 0.05 mg/ml collagenase type I, 0.025 mg/ml hyaluronidase, all from Sigma-Aldrich, St. Louis, MO; 0.01 mg/ml DNase I from Boehringer Mannheim, Ridgefield, CT) for 2 hours at 37°C with occasional shaking. The resultant cells were washed and passed through a Ficoll gradient to eliminate dead cells. Tumor infiltrating lymphocytes (TILs) were then analyzed by flow cytometry for the expression of CD4, CD8, and CD25 markers. T regulatory cells (T_reg_; Foxp3^+^) were analyzed using the anti-mouse Foxp3 staining kit (eBioscience). The same number of cells (based on side-scatter and forward-scatter analyses) was acquired in all samples. Anti-CD45.2 antibody was used to selectively exclude CD45^−^ tumor cells from analysis. For intracellular IFN-γ analysis, TILs were stimulated with PMA and Ionomycin for 8 hours in the presence of Brefeldin A.

### *In vivo* T lymphocyte subset depletion

Mice were depleted of lymphocyte subsets using ascites monoclonal antibodies (mAb) obtained from the American Type Culture Collection (ATCC, Manassas, VA), 53-6.72 for CD8^+^ T cells and GK1.5 for CD4^+^ T cells as described. Briefly, vaccinated mice were injected intraperitoneally (i.p.) with isotype control IgG, anti-CD8 or anti-CD4 mAb immediately preceding tumor challenge and every 4 days subsequently for a total of five injections.

### Carcinogenesis

Six-week-old male Balb/c mice received antioxidant-free laboratory chow for 2 weeks prior to the carcinogenesis regimen. At experimental week 1, a single dose of 3-methylcholanthrene (15 µg/g body weight) was administered *i.p.* dissolved in corn oil. On weeks 3–8, mice received six weekly *i.p.* doses of butylated hydroxytoluene (BHT) dissolved in corn oil. The first dose was 150 µg/g mouse weight and subsequent doses were 200 µg/g mouse weight. On weeks 4, 6 and 8, mice were vaccinated (subcutaneously, left flank) with HBSS alone or ESC/STO-GM. Mice were euthanized 18 weeks after administration of the single dose of 3-methylcholanthrene and the lungs were resected and inflated at a pressure of 15 cm with 4% buffered formalin. Surface tumors were enumerated by inspection under 5× magnification. In three animals of each group, whole lung serial sections (5 µm thickness) were made and 1 in every 20 sections (100 µm apart) were stained with H&E. Stained sections were digitized using a Nikon Cool Pix camera attached to a Nikon Dissection microscope (5× magnification). Each digitized image was quantified by measuring the total cross-sectional area of the lung and that of the tumor lesions using MetaMorph Version 6.1 (Universal Imaging Corporation, Sunnyvale, CA) with the help of an mm^2^ reference grid developed in our laboratory. The lesions were enumerated and classified by histopathological examination under a Nikon light microscope at 40×, 100× and 200× magnification. The tumorigenesis index was determined by calculating the ratio between the total area of tumors and the total tissue area of lungs. Two histopathologists examined the material and both were unaware of the experimental groups from which the animals were derived.

### Statistical analysis

StatView version 5.0.1 software (Windows version; SAS Institute, Cary, NC, USA) or GraphPad Prism 5.0 software (GraphPad Prism Software, Inc., La Jolla, CA, http://www.graphpad.com) was used for all statistical analyses. Comparisons between groups were done by Student's *t* test or one-way analysis of variance tests (ANOVA), where appropriate. Survival curves were analyzed using the log-rank test. For all tests, statistical significance was assumed where *p*<0.05.

## Supporting Information

Figure S1 **Flow cytometric analysis showing the intracellular expression of Sox2, Oct3/4, SSEA1, SSEA4 and Nanog – pluripotent stem cell markers - in undifferentiated murine ES-D3 cells.** Numbers in the quadrants represent the percentages of each subpopulation.
(TIF)

Figure S2 **ESC vaccination delays** ***in vivo*** **melanoma outgrowth and induces melanoma-specific, Th1-mediated cytokine response in CD8^+^ T cells.** (**A**) C57BL/6 mice (8/group) were immunized twice (days 0 and 14) with HBSS (control), or irradiated 1×10^6^ ESC+irradiated 1×10^6^ STO-GM, or irradiated 1×10^6^ STO-GM cells alone s.c. in the right flank prior to s.c. challenge with B16 melanoma cells on day 21. Tumor growth was measured by calipers every 2nd or 3rd day and tumor volumes were plotted as indicated. The data represent the average tumor volumes of 8 mice/group and are representative of three independent experiments. Error bars represent mean ± SEM. (**B**) C57BL/6 mice (6/group) were immunized twice (days 0 and 14) with HBSS (control) or irradiated 1×10^6^ ESC+irradiated 1×10^6^ STO-GM, s.c. in the right flank. Ten days after the boost, mice were euthanized and spleens were removed. Splenocytes from vaccinated and control mice were co-cultured with B16 lysate (50 µg/ml) for an additional 4 days. Effectors were harvested and stimulated for 4 hours with PMA (50 ng/ml) and ionomycin (500 ng/ml) in the presence of Brefeldin A (1 µl/ml). After restimulation, effectors were harvested, Fc receptors were blocked, and stained for surface expression of CD4, CD8 and intracellular expression of cytokines and analyzed by flow cytometry. Dot plots showing TNF-α and IFN-γ expression in CD8^+^ cells in splenocyte cultures obtained from control and ESC/STO-GM vaccinated mice. Numbers in quadrants represent the percentages of each subpopulation.
(TIF)
