# Supplementary figures and images for: Vaccination with Embryonic Stem Cells Protects against Lung Cancer: Is a Broad-Spectrum Prophylactic Vaccine against Cancer Possible?
Source: PLoS One. 2012 Jul 31;7(7):e42289. doi: 10.1371/journal.pone.0042289 (PMC3409174; doi:10.1371/journal.pone.0042289)

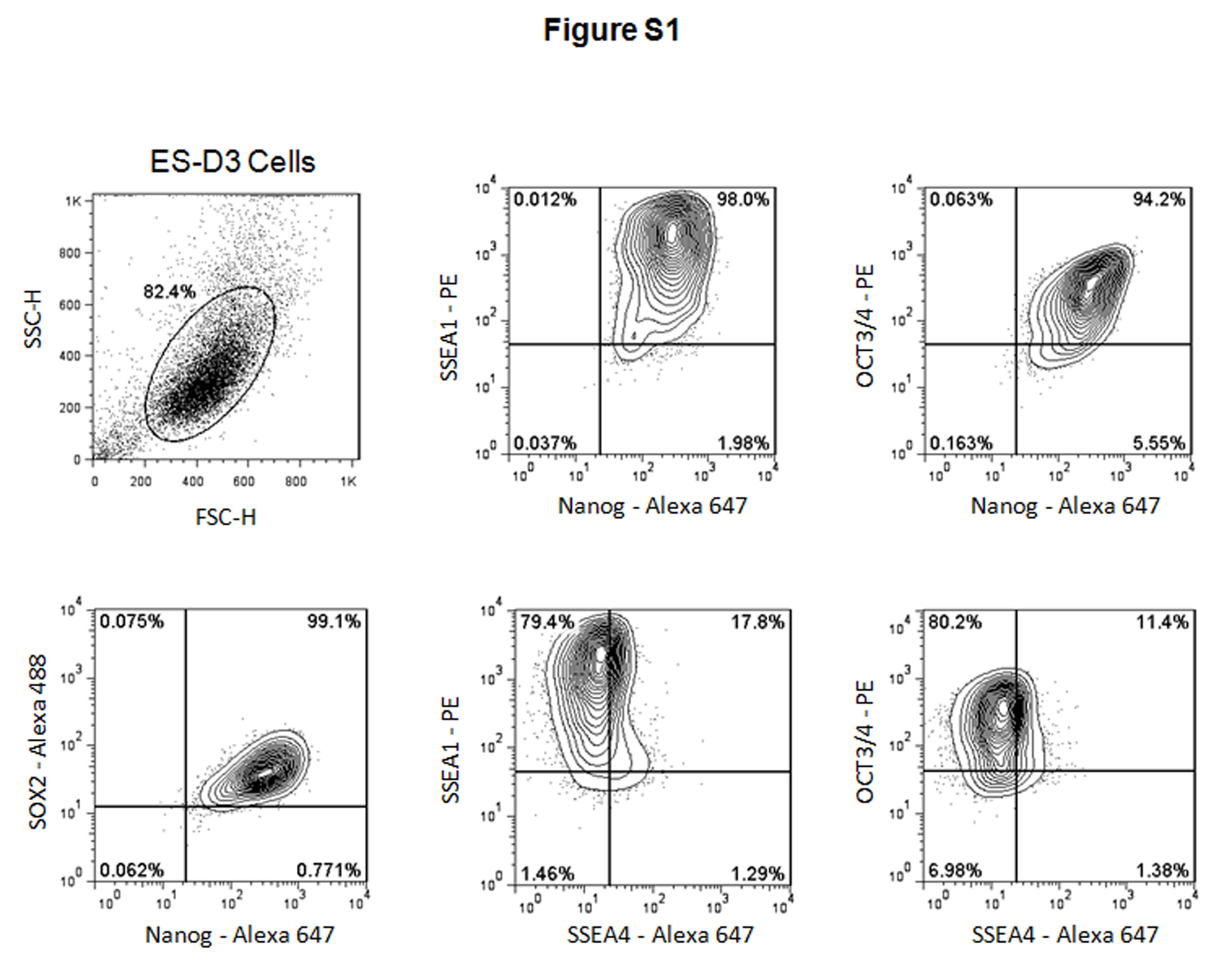

Supplement: Figure S1 — Flow cytometric analysis showing the intracellular expression of Sox2, Oct3/4, SSEA1, SSEA4 and Nanog – pluripotent stem cell markers - in undifferentiated murine ES-D3 cells. Numbers in the quadrants represent the percentages of each subpopulation. (TIF) [file pone.0042289.s001.tif]

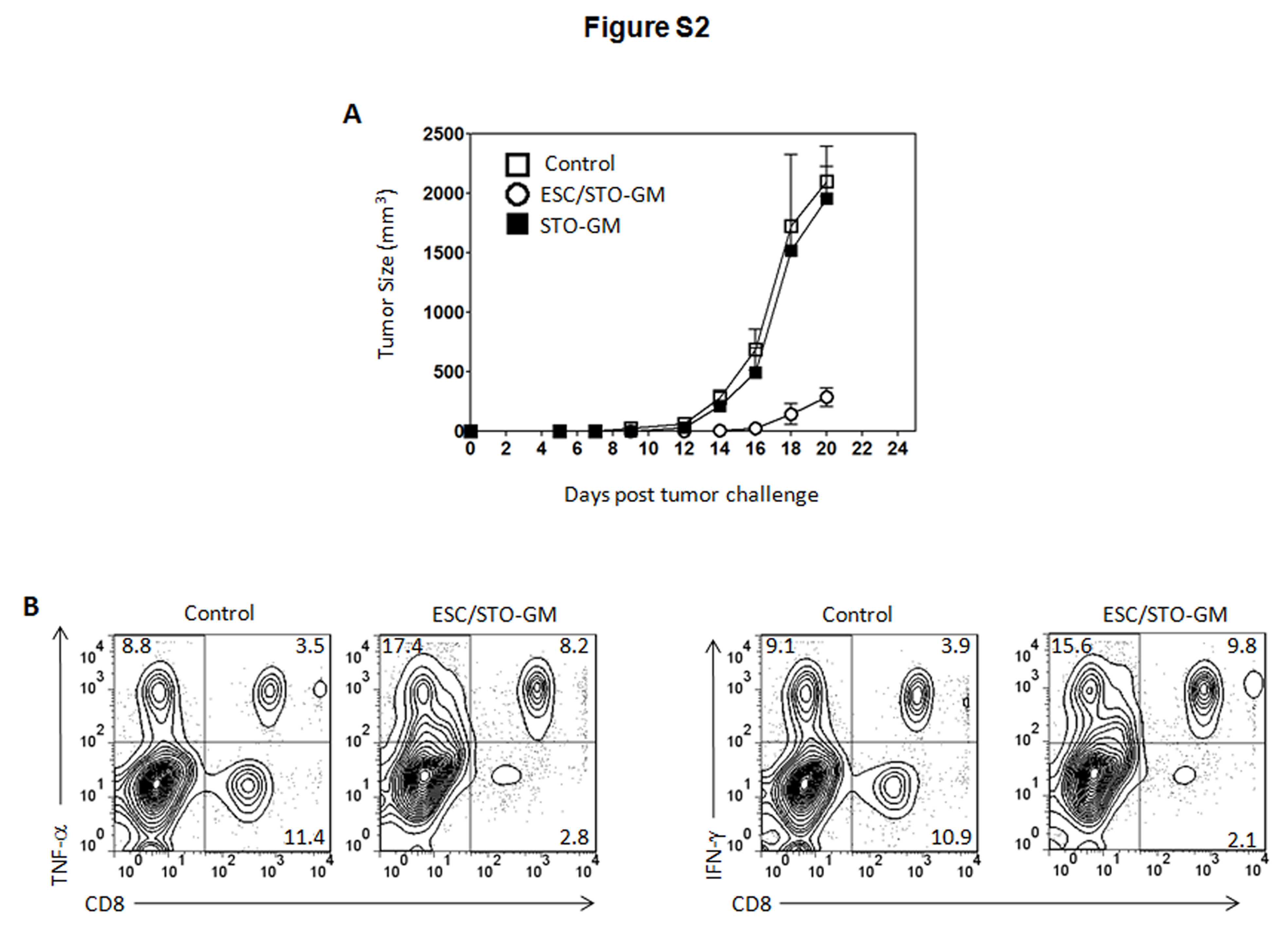

Supplement: Figure S2 — ESC vaccination delays in vivo melanoma outgrowth and induces melanoma-specific, Th1-mediated cytokine response in CD8+ T cells. (A) C57BL/6 mice (8/group) were immunized twice (days 0 and 14) with HBSS (control), or irradiated 1×10^6^ ESC+irradiated 1×10^6^ STO-GM, or irradiated 1×10^6^ STO-GM cells alone s.c. in the right flank prior to s.c. challenge with B16 melanoma cells on day 21. Tumor growth was measured by calipers every 2nd or 3rd day and tumor volumes were plotted as indicated. The data represent the average tumor volumes of 8 mice/group and are representative of three independent experiments. Error bars represent mean ± SEM. (B) C57BL/6 mice (6/group) were immunized twice (days 0 and 14) with HBSS (control) or irradiated 1×10^6^ ESC+irradiated 1×10^6^ STO-GM, s.c. in the right flank. Ten days after the boost, mice were euthanized and spleens were removed. Splenocytes from vaccinated and control mice were co-cultured with B16 lysate (50 µg/ml) for an additional 4 days. Effectors were harvested and stimulated for 4 hours with PMA (50 ng/ml) and ionomycin (500 ng/ml) in the presence of Brefeldin A (1 µl/ml). After restimulation, effectors were harvested, Fc receptors were blocked, and stained for surface expression of CD4, CD8 and intracellular expression of cytokines and analyzed by flow cytometry. Dot plots showing TNF-α and IFN-γ expression in CD8+ cells in splenocyte cultures obtained from control and ESC/STO-GM vaccinated mice. Numbers in quadrants represent the percentages of each subpopulation. (TIF) [file pone.0042289.s002.tif]
